# A Virtual World Versus Face-to-Face Intervention Format to Promote Diabetes Self-Management Among African American Women: A Pilot Randomized Clinical Trial

**DOI:** 10.2196/resprot.3412

**Published:** 2014-10-24

**Authors:** Milagros C Rosal, Robin Heyden, Roanne Mejilla, Roberta Capelson, Karen A Chalmers, Maria Rizzo DePaoli, Chetty Veerappa, John M Wiecha

**Affiliations:** ^1^University of Massachusetts Medical SchoolDivision of Preventive and Behavioral MedicineWorcester, MAUnited States; ^2^Heyden Ty, LLCAlameda, CAUnited States; ^3^Department of Family MedicineBoston Medical Center and Office of Academic AffairsBoston University School of MedicineBoston, MAUnited States; ^4^Department of Family MedicineSection of Endocrinology, Diabetes and Nutrition, Boston Medical CenterBoston University School of MedicineBoston, MAUnited States

**Keywords:** African Americans, clinical trials, feasibility, health behavior, health disparities, minority health, technology, type 2 diabetes, virtual systems, randomized clinical trial

## Abstract

**Background:**

Virtual world environments have the potential to increase access to diabetes self-management interventions and may lower cost.

**Objective:**

We tested the feasibility and comparative effectiveness of a virtual world versus a face-to-face diabetes self-management group intervention.

**Methods:**

We recruited African American women with type 2 diabetes to participate in an 8-week diabetes self-management program adapted from Power to Prevent, a behavior-change in-person group program for African Americans with diabetes or pre-diabetes. The program is social cognitive theory–guided, evidence-based, and culturally tailored. Participants were randomized to participate in the program via virtual world (Second Life) or face-to-face, both delivered by a single intervention team. Blinded assessors conducted in-person clinical (HbA1c), behavioral, and psychosocial measurements at baseline and 4-month follow-up. Pre-post differences within and between intervention groups were assessed using t tests and chi-square tests (two-sided and intention-to-treat analyses for all comparisons).

**Results:**

Participants (N=89) were an average of 52 years old (SD 10), 60% had ≤high school, 82% had household incomes <US $30,000, and computer experience was variable. Overall session attendance was similar across the groups (6.8/8 sessions, *P*=.90). Compared to face-to-face, virtual world was slightly superior for total activity, light activity, and inactivity (*P*=.05, *P*=.07, and *P*=.025, respectively). HbA1c reduction was significant within face-to-face (−0.46, *P*=02) but not within virtual world (−0.31, *P*=.19), although there were no significant between group differences in HbA1c (*P*=.52). In both groups, 14% fewer patients had post-intervention HbA1c ≥9% (virtual world *P*=.014; face-to-face *P*=.002), with no significant between group difference (*P*=.493). Compared to virtual world, face-to-face was marginally superior for reducing depression symptoms (*P*=.051). The virtual world intervention costs were US $1117 versus US $931 for face-to-face.

**Conclusions:**

It is feasible to deliver diabetes self-management interventions to inner city African American women via virtual worlds, and outcomes may be comparable to those of face-to-face interventions. Further effectiveness research is warranted.

**Trial Registration:**

ClinicalTrials.gov NCT01340079; http://clinicaltrials.gov/show/NCT01340079 (Archived by WebCite at http://www.webcitation.org/6T2aSvmka).

## Introduction

Type 2 diabetes is a complex chronic illness requiring continuing medical care and, ideally, patient adherence to numerous behavioral recommendations for self-management (ie, prescriptions for dietary change, physical activity, weight reduction, blood glucose self-monitoring, smoking cessation, and medication intake) [1] with the goal of achieving glucose control and preventing diabetes complications. Suboptimal control of diabetes places individuals at higher risk for diabetes complications [2].

There are considerable disparities in diabetes risk and outcomes in the population, with African Americans demonstrating among the highest diabetes prevalence and related morbidity and mortality [3,4]. Projected increases in incidence of diabetes may fuel even greater disparities in the future [3]. The traditional medical model involving repeated face-to-face visits over time may represent barriers to diabetes management, especially among underserved populations such as African Americans. Competing family responsibilities, distance to services, transportation difficulties and cost, cost of time away from work and other responsibilities, and difficulties accessing care [5] are among the reasons for limited participation in treatment among patients and may contribute to poor outcomes among African Americans.

With increased penetration rates of Internet use, at 81% in 2013 [6] (up from 71.7% in only 2011) [7], researchers have investigated the impact of delivering behavioral interventions via the Web. The Internet offers alternatives to the challenges typically associated with face-to-face lifestyle interventions through its potential for increased access to specialized behavior change experts, convenience to patients, and potentially lowered costs. However, while online alternatives show promising improvement in health behaviors and glycemic control, effect sizes have been small [8-11]. Limited human interactivity and engagement have been hypothesized as contributors to the small effect sizes [12,13]. In contrast, Virtual world technologies are potentially more suitable environments for supporting diabetes self-management programming. Through the use of three-dimensional (3D) environments that depict real places and avatars that represent people, virtual world environments offer opportunities for interaction, intense engagement, and opportunities for scripted immersive experiences, simulations, role-playing, and constructivist experiences, all important facilitators of active learning [14,15]. The use of virtual world environments continues to increase. There were 1772 million registered virtual world accounts in 2011, with 27 million users registered in Second Life alone [16,17]. The potential of virtual world environments for implementing or supplementing diabetes care interventions has been noted [18], but there is little evidence for the feasibility and potential effectiveness of such an approach [19].

This pilot study examined the feasibility of delivering a group-based diabetes self-management intervention via a virtual world environment (Second Life) and explored the potential effectiveness of the virtual world-based intervention, compared to a traditional face-to-face intervention, on self-management behaviors and glucose levels.

## Methods

### Design

A randomized clinical trial design was used. A detailed description of the study methods has already been published [20]. The Institutional Review Boards at Boston Medical Center and the University of Massachusetts Medical School approved the trial, and all participants provided written consent prior to participating in the trial.

### Participants

Study participants were African American women identified from the medical record data warehouse at Boston Medical Center and affiliated community health centers as having a diagnosis of type 2 diabetes, age ≥18 years, English-speaking, HbA1c>8 at their last outpatient visit (within the previous 12 months), and excluded for medical conditions for which the intervention diet and physical activity would be contraindicated (ie, ulcerative colitis, renal failure, complications following abortion and ectopic and molar pregnancies, angina pectoris, and other forms of unstable ischemic heart disease and other conditions precluding brisk walking). Identified patients were mailed a letter to inform them about the study, to announce a phone call from study staff, and to provide the option to call in or opt out. The staff made up to five calls per patient (on different days and times). Those patients reached were informed about the study (ie, comparison of two formats for delivering a diabetes self-management intervention) and screened for interest and final eligibility (ie, self-reported ability to view a computer screen without difficulty, ability to read, no use of glucocorticoid therapy, no current participation in a weight loss program, and availability for weekly meetings). Fully eligible and interested women were invited to participate and scheduled for an in-person enrollment visit at the Boston Medical Center General Clinical Research Unit. At this visit, participants provided written informed consent and completed baseline assessments.

### Randomization

Upon completion of baseline assessment measures, participants were randomized to either the virtual world-based intervention or the face-to-face intervention. Randomization was stratified by age and hemoglobin A1C measured at baseline using a block randomization scheme with a block size of 4, developed by StudyTRAX software (v3.0.0103).

### Intervention Conditions

The intervention protocol was similar in both conditions, adapted from the Centers for Disease Control/National Institutes of Health program “Power to Prevent” [21], a widely available social cognitive theory-guided [14], evidence-based, and culturally appropriate behavior-change curriculum designed for delivery to African American groups with diabetes or pre-diabetes via face-to-face group sessions. The intervention sought to enhance diabetes knowledge, optimize attitudes toward diabetes self-management (ie, self-efficacy, outcome expectations), and develop behavioral self-management skills (eg, goal setting, tracking self-management behaviors and glucose levels, problem solving) to facilitate changes in diet, physical activity, blood glucose self-monitoring, and medication adherence. The first session used an individual format followed by eight weekly 90-minute group sessions (group size was 8-9 participants). A single intervention team (a registered dietitian who is a certified diabetes educator, and a nurse practitioner), trained in behavioral counseling and motivational interviewing principles, delivered all sessions in the virtual world environment or face-to-face using the same protocol consisting of a detailed intervention manual and materials (intervention delivery methods are described in greater detail elsewhere) [20]. Intervention fidelity was monitored, and providers were given feedback on behavioral counseling process and content. Participants in both conditions received a two-session computer training and were provided with an Internet-enabled laptop computer upon training completion (Internet access was standardized by providing high-speed 4th generation wireless modems to all participants).

The virtual world-based intervention was delivered in a mock open-air virtual world forum designed and programmed especially for the intervention with appropriate structures and visuals/displays (eg, food exhibits, confidence ruler, a ring of screens, exercise facilities). Participants were asked to log in 30 minutes prior to each session in order to troubleshoot connection or sound problems. A triage system to provide technical support as needed through the session was used. The face-to-face intervention took place in a large conference room at Boston Medical Center. All face-to-face participants received transportation vouchers to facilitate attendance.

### Measures and Data Collection

Trained staff, blinded to study condition, conducted assessments at baseline and at 4-month follow-up. Clinical assessments included a non-fasting blood sample for HbA1c assays (specimens were analyzed at the Boston Medical Center laboratories) and measures of blood pressure, height, weight, and waist circumference using standard protocols [20]. At each baseline and follow-up assessment, two telephone-administered unannounced 24-hour recalls assessed diet and physical activity [22,23], blood glucose self-monitoring, and medication adherence. Survey measurements were verbally administered and included measures of depressive symptoms [24], self-efficacy for diabetes management [25], health literacy [26], social support [27], perceived stress [28], quality of life [29], demographics, and other characteristics, including baseline experience with computers and the Internet and post-intervention participant satisfaction. Intervention implementation costs (costs that would be incurred if the intervention were to be implemented outside the context of the research project) were tracked, including staff time, facilities, materials, and set-up (Second Life) for all sessions.

### Data Analysis

An intent-to-treat approach was used to compare the virtual world versus face-to-face groups. Feasibility was assessed by comparing the attendance rate by session and the mean number of sessions attended within each arm. Implementation costs were also considered in determining feasibility. Cost estimates were based on expenditures from the trial and excluded the cost of equipment for participants in the virtual world groups. All primary and secondary outcomes were assessed for pre-post differences within each respective arm. The pre-post differences were then compared between the two arms. Differences between continuous variables were assessed using *t* tests. Binomial tests were used for categorical outcomes. Non-parametric tests were applied as appropriate. Analysis of potential mediators was conducted using the taxonomy and recommendations of Zhao [30]. Analyses were conducted in SAS 9.1 and R (version i386 2.153), all comparisons were two-sided and *P*<.05 was considered statistically significant.

We performed bivariate analysis of baseline characteristics to determine whether randomization achieved balance in both treatment groups across all characteristics. The results revealed a statistically significant difference in the proportion of participants with systolic blood pressure greater than 130 mmHg. While multivariate adjustment eliminated the statistical significance of systolic blood pressure at baseline, adjustment did reveal an imbalance in insulin use between the two treatment groups. Using a general linear regression to evaluate the association of insulin use on the pre-post change in HbA1c, we found that it did not have a statistically significant impact and did not affect our assessment of no difference between the virtual world group versus the face-to-face group.

## Results

**Figure 1:**
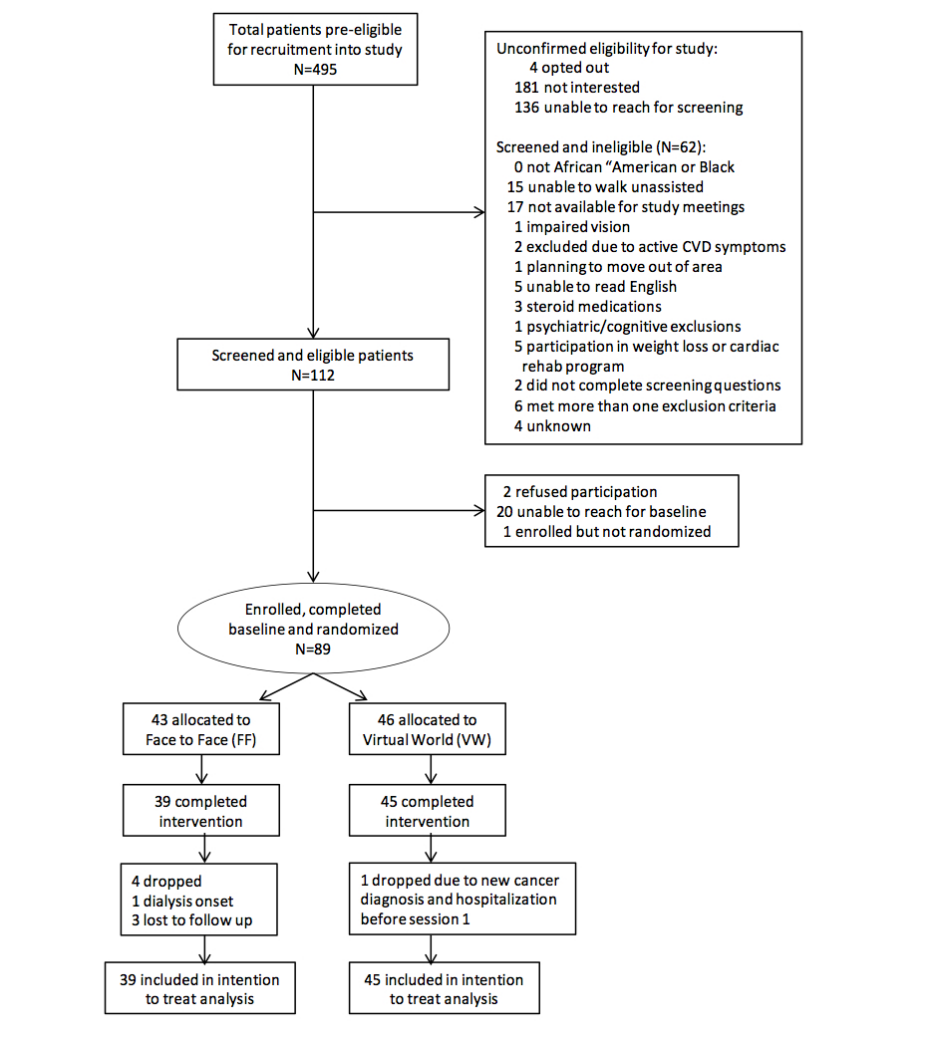
Flowchart of participant screening, recruitment, randomization, and retention.

Of the 494 patients who were deemed pre-eligible based on medical records data (age 18 or greater, English-speaking, type 2 diabetes diagnosis, last HbA1c>8 within previous 12 months), it was not possible to determine the eligibility of 321 patients for reasons listed in Figure 1. Of the 174 (35%) who were reached for telephone screening, 62 (36%) were ineligible and 112 (64%) were eligible. From these 112 patients with known eligibility, 89 (79%) were enrolled and randomized, 46 of them to the virtual world intervention and 43 to the face-to-face intervention.

Table 1 summarizes demographic and baseline characteristics of participants in the trial: average age was 52 years (SD 10) and 90% of participants were over the age of 40; 60% had a high school education or lower; 82% reported a household income of US $30,000 or less; and experience with computers was variable. The single statistically significant difference between the virtual world and face-to-face groups at baseline was the proportion of participants with systolic blood pressure greater than 130 mmHg. A greater proportion of participants in the face-to-face group had elevated systolic pressure compared to those in the virtual world group (18% vs 10%, *P*=.04).

**Table 1:** Sample characteristics at baseline.

			All subjects (N=89)	virtual world (n=46)	Face-to-face (n=43)	*P*
**Demographic characteristics**						
	**Age in years, mean (SD), IQR**		52 (10), 49-58	53 (10), 49-59	52 (11), 48-57	.77
		18-40, n (%)	9 (10.1)	6 (13.0)	3 (7.0)	.49^a^
		>40, n (%)	80 (89.9)	40 (87.0)	40 (93.0)	
	**Marital status, n (%)**					
		Single (never married)	45 (50.6)	27 (58.7)	18 (41.9)	
		Married or living with partner	19 (21.4)	9 (19.6)	10 (23.3)	
		Separated, divorced, or widowed	25 (28.0)	10 (21.7)	15 (34.8)	
	**Education in years, mean (SD), IQR**		13.1 (2.2), 12-16	13.3 (2.3), 12-16	12.8 (2.0), 12-14	.30
		<High school, n (%)	16 (18.0)	7 (15.2)	9 (20.9)	.44
		High school graduate, n (%),	37 (41.6)	17 (37.0)	20 (46.5)	
		Vocational/Assoc degree, n (%)	13 (14.6)	9 (19.6)	4 (9.3)	
		≥College, n (%)	23 (25.8)	13 (28.3)	10 (23.3)	
	**Work status, n (%)**					
		Working full or part-time	32 (36.0)	13 (28.3)	19 (44.2)	.12
		Not working	57 (64.0)	33 (71.7)	24 (55.8)	
	**Household income, n (%)**					
		≤$10,000	28 (31.5)	16 (34.8)	12 (27.9)	.82
		$10,000-$30,000	45 (50.5)	23 (50.0)	22 (51.2	
		≥$30,000	10 (11.2)	6 (13.0)	4 (9.3)	
		Declined	6 (6.7)	1 (2.2)	5 (11.6)	
	**Insurance, n (%)**					
		Public/no insurance	68 (76.4)	37 (80.4)	31 (72.0)	.35
		Private	21 (23.6)	9 (19.6)	12 (27.9)	
	**Health literacy (confidence filling out medical forms by herself), n (%)**					
		Extremely	61 (72.6)	37 (82.2)	24 (61.5)	.19^a^
		Quite a bit	6 (7.1)	1 (2.2)	5 (12.8)	
		Somewhat	10 (11.9)	4 (8.9)	6 (15.4)	
		A little	5 (5.9)	2 (4.4)	3 (7.7)	
		Not at all	2 (2.4)	1 (2.2)	1 (2.6)	
**Computer experience**						
	Hrs/wk using a computer, mean (SD)		13 (17)	11 (12)	15 (21)	.39
	**Home Internet access, n (%)**					
		Yes	61 (68.54)	34 (73.91)	27 (62.79)	.26
		No	28 (31.46)	12 (26.09)	16 (37.21)	
	**Able to start and shut down computer on her own, n (%)**					
		Not at all	5 (5.62)	3 (6.52)	2 (4.65)	.59^a^
		With a lot of help	3 (3.37)	1 (2.17)	2 (4.65)	
		With a little bit of help	18 (20.22)	7 (15.22)	11 (25.58)	
		Without help	63 (70.79)	35 (76.09)	28 (65.12)	
	**Able to create and send email on her own, n (%)**					
		Not at all	13 (14.61)	5 (10.87)	8 (18.60)	.18^a^
		With a lot of help	8 (8.99)	3 (6.52)	5 (11.63)	
		With a little bit of help	20 (22.47)	8 (17.39)	12 (27.91)	
		Without help	48 (53.93)	30 (65.22)	18 (41.86)	
	**Able to go online on the Internet on her own, n (%)**					
		Not at all	9 (10.11)	2 (4.35)	7 (16.28)	.16
		With a lot of help	8 (8.99)	5 (10.87)	3 (6.98)	
		With a little bit of help	11 (12.36)	4 (8.70)	7 (16.28)	
		Without help	61 (68.54)	35 (76.09)	26 (60.47)	
	**Use Internet to search for health information, n (%)**					
		Never	33 (37.08)	13 (28.26)	20 (46.51)	.19^a^
		Rarely	8 (8.99)	4 (8.70)	4 (9.31)	
		Occasionally	30 (33.71)	20 (43.48)	10 (23.26)	
		Often	18 (20.22)	9 (19.57)	9 (20.93)	
	Use Second Life (yes)		3 (3.37)	0	3 (6.98)	.11
**Health characteristics, n (%)**						
	**HbA1c**					
		<7%	7 (7.9)	4 (8.7)	3 (7.0)	1.0
		7-7.9%	14 (15.7)	7 (15.2)	7 (16.3)	
		8-8.9%	13 (14.6)	7 (15.2)	6 (13.9)	
		≥9.0%	55 (61.8)	28 (60.9)	27 (62.8)	
	Insulin usage		44 (49.4)	21 (45.7)	23 (53.5)	.46
	**BMI (kg/m** ^**2**^ **), n (%)**					
		Normal (<25)	4 (4.5)	2 (4.4)	2 (4.7)	.82
		Overweight (25-29.9)	18 (20.2)	8 (17.4)	10 (23.3)	
		Obese I (30-34.9)	27 (30.3)	13 (28.3)	14 (32.6)	
		Obese II (35-39.9)	21 (23.6)	11 (23.9)	10 (23.3)	
		Obese III (≥40)	19 (21.3)	12 (26.1)	7 (16.3)	
	Waist circumference >35 in, n (%)		80 (89.9)	41 (89.1)	39 (90.7)	1.0^a^
	Systolic blood pressure >130, n (%)		28 (31.5)	10 (21.7)	18 (41.9)	.04
	Diastolic blood pressure >80, n (%)		48 (53.9)	22 (47.8)	26 (60.5)	.23
	Total cholesterol >200, n (%)		29 (32.6)	13 (28.3)	16 (37.2)	.37

^a^ *P* value derived from Freeman-Halton extension of Fisher’s exact test.

### Feasibility of the Virtual World-Based Intervention

Overall session attendance was similar across the two interventions, with an average 6.8 sessions (SD 1.8) among WV participants and 6.8 (SD 1.7) among face-to-face participants (*P*=.9). However, a significant difference was observed in the rate of completion of Session 1. Compared to face-to-face participants, fewer virtual world participants completed this session (78%, 36/46 vs 95%, 41/43, *P*=.02, respectively). There was also a slight although non-significant difference in the proportion of participants completing sessions 1-3 in the virtual world group (63%, 29/46) versus the face-to-face group (77%, 33/43) (*P*=.16).

Overall participant retention rate was 94% for clinical and psychosocial assessments, and 93% for telephone-based assessments, and attrition was lower in the virtual world group (1 participant, or 2%) compared to the face-to-face group (4 participants, or 9%) (*P*=.19). The one drop-out in the virtual world group was due to a new cancer diagnosis and unexpected hospitalization and occurred prior to session 1. Reasons for drop-out in the face-to-face group included dialysis onset and loss to follow-up (2 of these 4 participants attended Session 1).

### Health Outcomes

Results from intention-to-treat analyses are shown in Table 2. There were improvements associated with both the virtual world and the face-to-face intervention conditions. Analysis of change from baseline to 4-month follow-up within the groups revealed a non-statistically significant 3.2% reduction in HbA1c in the virtual world group (*P*=.186) and a significant 4.9% reduction in HbA1c in the face-to-face group (*P*=.019). However, no significant differences between the groups were detected (*P*=.52). There was also a statistically significant within group decrease in the percentage of participants with HbA1c ≥9% in both groups, with 14% fewer participants in each group having a HbA1c value above 9.0% (*P*=.014 and *P*=.002, for virtual world and face-to-face groups, respectively), with no significant differences between the groups (*P*=.493). No significant within or between-group changes were observed in measures of blood pressure, total cholesterol, waist circumference, and Body Mass Index (BMI).

### Behavioral Outcomes

Participants in the virtual world group experienced an 18.4% within-group increase in total physical activity and a significant 8% decrease in inactivity (*P*=.10 and *P*=.04, respectively), whereas participants in the face-to-face group experienced a 22.5% reduction in their total physical activity. There was a marginally significant between difference in total physical activity, with marginally superior effects for the virtual world compared to face-to-face group on total activity, light activity, and inactivity (*P*=.05, *P*=.07, and *P*=.025, respectively). The proportion of participants not adhering to blood glucose self-monitoring dropped by half in both groups (*P*=.001 and *P*=.002 for virtual world and face-to-face, respectively), with no significant between-group differences for this outcome. No significant within or between-group differences were observed for dietary outcomes of interest (ie, total calories, percent calories of saturated fat, fiber or dietary quality as measured by the Alternate Healthy Eating Index). Medication adherence decreased in the face-to-face group with 8.6% fewer participants reporting that they adhered to all medications as prescribed (*P*=.035), whereas there was a 1.2% increase in the virtual world group, although no differences between the groups with regards to change in self-reported medication adherence (*P*=.298).

### Psychosocial Outcomes

Depression symptom scores and mental health functioning (as measured by the Short-Form survey [SF-12]) were marginally improved in the face-to-face condition only (*P*=.053 and *P*=.062, respectively), and there were between-group differences for depression symptom score pre-post change (*P*=.051). Improvements in self-efficacy for diabetes self-management were observed in both groups (*P*<.001), and there were no differences in self-efficacy improvements between the groups (*P*=.268). No within or between-group differences were observed for perceived stress or social support.

### Mediation Analysis

We evaluated the potential mediation effects of select behavioral and psychosocial outcomes and found insufficient evidence of a mediation effect on HbA1c levels for changes in total calories consumed, total calories from saturated fat, total dietary fiber, alternate healthy eating index, diabetes self efficacy scores, inactivity levels, activity levels (household, light, and moderate activity), medication adherence post intervention, and blood glucose self-monitoring (data not shown).

### Intervention Costs

The per-participant cost of implementing the virtual world intervention was US $186.39 greater compared to cost of implementing the face-to-face intervention (US $1117 vs $931 for virtual world vs face-to-face, respectively). Expenses associated with health care personnel (eg, diabetes nurse educator, dietitian, administrative staff) and educational materials were the same between the two groups. However, the virtual world group required additional technical personnel who trained and provided technical assistance to participants and the intervention team during each session, contributing to 13% of the total cost per participant in that group.

### Participant Satisfaction

At the follow-up assessment, 97% of face-to-face participants agreed/strongly agreed with the statement “If I had a choice, I would attend diabetes sessions face to face rather than on a computer”, while 80% of virtual world participants agreed/strongly agreed with the statement “If I had a choice, I would attend diabetes sessions on Second Life rather than face-to-face at BMC or my health center” (*P*=.490). However, there were no differences between the groups with regard to whether they would recommend their program to other people; 100% of virtual world participants agreed/ strongly agreed with the statement “I would recommend other people to attend diabetes education sessions given in Second Life,” and 97% of face-to-face participants agreed/ strongly agreed with the statement “I would recommend other people to attend diabetes education sessions given face to face at BMC or a health center” (*P*=1.0).

**Table 2:** Within group and between group comparisons for the virtual world and face-to-face intervention conditions (*P* values for continuous variables derived from Student’s *t* test of pre-post differences, unless otherwise noted).

Variable			Face-to-face					Virtual world					
			Baseline (n=43)	Post (n=39)	Baseline/ follow-up difference	% change	Within group, *P*	Baseline (n=46)	Post (n=45)	Baseline/ follow-up difference	% change	Within group, *P*	Between group, *P*
**Clinical outcomes, mean (SD)**													
	**HbA1c**		9.4 (2)	8.9 (2)	-0.46	-4.9	.019	9.6 (2)	9.3 (2)	-0.31	-3.2	.186	.519
		HbA1c<9^a,b^, %	37.2	51.3			.002	39.1	53.3			.014	.493
	Systolic BP, mmHg		126.0 (15)	126.0 (17)	0.05	0.04	.808	120.5 (13)	122.3 (16)	1.81	1.5	.233	.609
	Diastolic BP, mmHg		80.4 (11)	78.7 (9)	-1.64	-2.0	.733	79.4 (9)	80.1 (10)	0.72	0.9	.600	.675
	Cholesterol		194.6 (42)	191.1 (40)	-3.50	-1.8	.186	187.8 (49)	186.9 (45)	-0.90	-0.5	.971	.412
	BMI		34.4 (8)	33.4 (6)	-1.00	-2.9	.912	36.4 (8)	36.1 (8)	-0.30	-0.8	.134	.200
	Waist circumference (in)		110.0 (17)	107.2 (15)	-2.80	-2.5	.631	113.1 (17)	112.1 (16)	-1.03	-0.9	.415	.837
**Behavioral variables** (weekday)													
	**Self-reported diabetes medication adherence, %**												
		All prescribed diabetes medications^a,b^	88.1	79.5			.035	86.9	88.1			.488	.2984
	**Dietary intake, weekday averages, mean (SD)**												
		Total calories, kcal	1377.0 (512)	1181.3 (443)	-195.67	-14.2	.023	1220.7 (518)	1136.0 (492)	-84.70	-6.9	.257	.255
		% calories from SFA	11.0 (4)	9.8 (4)	-1.17	-10.6	.162	10.7 (4)	9.9 (3)	-0.78	-7.3	.434	.544
		Fiber	11.4 (6)	13.1 (8)	1.63	14.2	.163	13.0 (7)	13.6 (7)	0.59	4.5	.627	.496
		Alternate Healthy Eating index	27.0 (9)	29.8 (9)	2.86	10.6	.121	29.4 (10)	30.6 (10)	1.16	3.9	.548	.469
	**Physical activity (PA), weekday averages, mean (SD)**												
		Total PA (MET-hr)	40.9 (32)	31.7 (29)	-9.20	-22.5	.196	36.5 (27)	43.2 (31)	6.70	18.4	.113	.050
		Total inactivity (MET-hr)	63.0 (13)	65.2 (13)	2.24	3.5	.269	65.0 (12)	60.0 (15)	-5.00	-7.7	.040	.025
		Household activity (MET-hr)	18.0 (16)	11.8 (11)	-6.20	-34.4	.013	16.0 (11)	14.0 (11)	-2.00	-12.5	.318	.140
		Light activity (MET-hr)	36.0 (24)	28.6 (29)	-7.40	-20.6	.311	33.0 (27)	39.2 (32)	6.24	18.9	.101	.071
		Moderate activity (MET-hr)	16.0 (20)	15.9 (28)	-0.15	-0.9	.650	16.0 (26)	19.5 (29)	3.50	21.9	.363	.472
		Median	8.0	8.3				6.0	8.3				
	**Blood glucose self-monitoring** ^a^ **, %**												
		No monitoring	35.0	15.0			.002	24.0	12.0			<.001	.895
**Psychosocial variables, mean (SD)**													
	Depressive symptoms (CES-D)		22.6 (9)	20.6 (8)	-2.01	-8.9	.053	19.8 (9)	20.5 (10)	0.71	3.6	.441	.051
	Perceived stress (PSS)		14.2 (7)	13.9 (8)	-0.28	-2.0	.263	14.2 (8)	15.1 (7)	0.87	6.1	.336	.139
	Physical functioning (SF-12 PCS)		41.7 (9)	43.9 (11)	2.18	5.2	.247	42.4 (10)	42.3 (11)	-0.08	-0.2	.813	.322
	Mental health functioning (SF-12 MCS)		47 (10)	50 (11)	3.74	8.0	.062	49 (11)	50.3 (12)	1.32	2.7	.385	.293
	Overall quality of life (SF-12 total score)		88.5 (13)	94 (14)	5.88	6.6	.022	91.3 (16)	92.6 (14)	1.24	1.4	.599	.113
	Social support		68.0 (20)	72 (20)	3.50	5.1	.261	69.5 (27)	67.7 (28)	-1.79	-2.6	.602	.256
	Diabetes self-efficacy		34.6 (7)	40 (7)	5.90	17.1	<.001	36.0 (9)	40.6 (7)	4.58	12.7	<.001	.268

^a^Within group differences determined by Fisher’s Exact test.

^b^Between group differences determined by Breslow-Day Test for Homogeneity.

**Figure 2:**
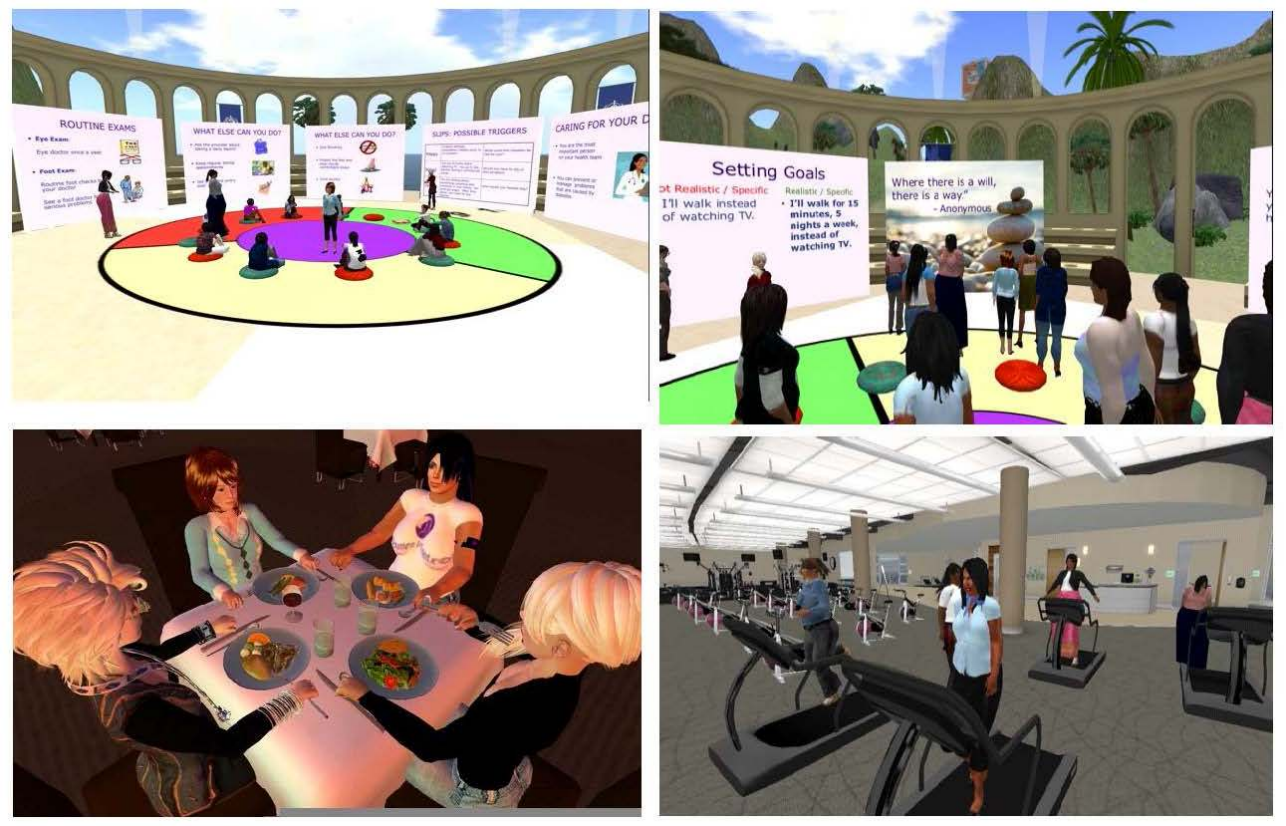
Pictures of the Virtual World participants engaged in various intervention sessions.

Figure 2 contains pictures of virtual world intervention sessions and edited clips of the sessions (see also Multimedia Appendix 1).

## Discussion

### Principal Findings

To our knowledge, this pilot randomized controlled trial (RCT) is the first study to compare the feasibility and potential comparative effectiveness of a virtual world-based versus an face-to-face-based diabetes self-management intervention. A previous publication reported on the feasibility of conducting individual virtual world-based visits with participants with diabetes, but the intervention did not include lifestyle modification, the study did not include a comparison condition, and metabolic outcomes were not reported [19]. Our study showed that it is feasible to deliver a virtual world group-based behavioral intervention originally designed for face-to-face delivery, to improve diabetes self-management among inner-city African American women. All participants who began the virtual world group completed the study, whereas 4 participants in the face-to-face condition did not.

Study findings show that the virtual world technology has tremendous potential for intervening and improving glucose control and diabetes self-management behaviors. Reductions in glucose levels were similar across both groups and were comparable to those of other group interventions [31]. Furthermore, HbA1c reductions were particularly significant among participants with the highest baseline glucose control (HbA1c≥9%).

Our study showed a marginal superiority of the virtual world intervention, compared to the face-to-face intervention, on physical activity (increase in total and light physical activity, and reduction in inactivity). A greater effect of the virtual world over the face-to-face intervention format on physical activity was also reported by Johnston et al [13]. In that study, the authors attributed this effect to the virtual world environment facilitating opportunities for the individual to initiate and practice healthy behaviors through an avatar with whom they identify. A study by Napolitano et al [32] that explored the usability of avatars for modeling weight loss behaviors provided additional support for the potential of virtual worlds for influencing diet and exercise behaviors. Two additional studies reported promising results from virtual world-based interventions for smoking cessation among rural teens [33,34]. Known as the Proteus effect, the practice of a new behavior by one’s avatar may influence the individual’s behavior in the real world [35].

The virtual world and face-to-face interventions were both comparable in terms of fostering blood glucose self-monitoring and enhancing diabetes management self-efficacy, and the face-to-face intervention was marginally superior compared to virtual world with regards to reducing depression symptoms. Social support interventions have improved depressive symptoms among people with diabetes, and it is possible that the face-to-face interaction influences participants in a different manner compared to virtual world interactions, potentially facilitating greater or a different type of social support [36].

### Strengths and Limitations

There were technical challenges in the virtual world intervention that affected completion of the first session. Additional technical difficulties occurred with decreasing frequency over the course of the intervention. The technical support during the virtual world sessions was necessary to assure that each participant was able to navigate, hear, and interact in the virtual world. The two most commonly encountered technical challenges were strength of Internet connection and sound problems (could not hear or could not speak). virtual worlds demand significant bandwidth as they process enormous amounts of data to render the 3D spaces, physical interactions, and sound that characterize these environments, and our chosen Internet service, provided via wireless modem, was unreliable in the participants’ neighborhoods (inadequate cell tower coverage). We devised a triage system troubleshooting these problems (re-positioning in the home, and checks for headphone, laptop, and Second Life preferences options). Undoubtedly, virtual world platforms are rapidly improving and becoming more accessible and technically efficient.

Despite these challenges, participants seemed to be equally satisfied and engaged in both intervention groups. Consistent with the high level of satisfaction reported by participants in both groups, attrition was remarkably low in this hard-to-reach group of African American women. A previous study of a virtual world-based versus face-to-face-based intervention that targeted weight loss in a non-minority, educated and more affluent group reported a 13% drop-out rate in both groups, with 5 virtual world participants reportedly dropping out within the first 2 weeks of the program for reasons associated with technical difficulties [13].

The per-participant cost of the virtual world intervention was 13% higher than that of the face-to-face intervention, with the excess cost related primarily to the need for technical support staff to train and support our participants, as the study participants had variable levels of computer experience. This study is unable to answer the question of whether the increased cost of the virtual world intervention outweighs its potential benefit; however, it does provide evidence of feasibility and preliminary evidence of effectiveness for future larger RCTs that can answer such questions. It is important to note that the cost of virtual world interventions should be expected to decrease over time with improved technologies and Internet access for the wider population.

A particular strength of the study was the use of the virtual world format with a socioeconomically disadvantaged sample of African American women. African Americans constitute a high risk group with a high prevalence of diabetes, diabetes complications, and mortality [3,4]. The sample was middle-aged with most women having a high school education or lower, low household income, and variable computer experience. Furthermore, the sample consisted of 79% of participants who were reached and for whom eligibility was known, supporting the representativeness of the sample and potential generalizability of the study findings. Prior studies of Internet-based interventions have included primarily young individuals. While it has been hypothesized that demographic differences (including age, ethnicity, income, and culture) could impact the effect of interventions [37], our study found that the virtual world intervention was feasible and had benefits for our African American sample. The generalizability of Internet-based interventions also has been questioned based on potential selection bias by which individuals with low computer or Internet literacy may refrain from participation, may be excluded by the study’s eligibility criteria, or may more easily drop out from these interventions. For example, the only prior pilot study comparing a virtual world-based versus face-to-face-based interventions for weight loss [13] recruited virtual world participants via print and online media and excluded participants who had no access to an Internet-connected computer (73% of participants held college or advanced degrees and had incomes above $75,000). In contrast, our study systematically recruited patients from community health centers and a large safety net hospital using electronic databases, and minimized exclusion criteria in an effort to provide accurate data on the feasibility and outcomes of the virtual world intervention for inner-city African American women. The fact that the sample had variable computer experience and most participants had no prior exposure to virtual world environments provides further support for the potential generalizability of virtual world-based behavioral interventions.

Additional study strengths include the parallel content and structure of the virtual world and the face-to-face interventions, and their delivery by a single provider team, with only the intervention format being different across groups, and the tracking of cost data for both groups. While the possibility of reduced care cost has been an argument for Web-based interventions, very few studies have compared the costs associated with the implementation of virtual world versus face-to-face.

### Conclusions

Future research is needed to test the comparative effectiveness of the virtual world and face-to-face interventions in larger, appropriately powered trials and with a longer follow-up. Future studies should also investigate characteristics of individuals who do best with each one of the two approaches. For example, it has been suggested that men may have a stronger experience of “presence” (ie, perceived realism, sense of being present) when being immersed in virtual world environments [38,39]. Furthermore, there is currently little understanding of potential mechanisms that facilitate health behavior change and adherence in virtual world environments. Future studies need to examine virtual world environment and avatar factors that facilitate health behavior change, including the degree to which one’s experience in the virtual world influences one’s behavior in the real world (Proteus effect) [35]. In 2010, the National Heart Lung and Blood Institute (NHLBI) convened a workshop entitled “Virtual Reality Technologies for Research and Education in Obesity and Diabetes”, which included behavioral and health researchers, technology experts, and representatives of the other National Institutes of Health institutes (National Cancer Institute, National Institute of Child Health and Human Development, National Institute of Diabetes and Digestive and Kidney Diseases, the NIH Office of Behavioral and Social Sciences Research, and the NIH Office of Research on Women’s Health). A report [40] from this workshop identified a number of research priorities, including the impact of using virtual reality technologies for fostering health-related behaviors and for extending the availability and capacity of health care providers (ie, “extended classrooms for diabetes education”). This study addressed both priorities.

## Multimedia Appendix 1

Link to video of Virtual World intervention sessions.

## Abbreviations

BMI: body mass index
BP: blood pressure
CES-D: Center for Epidemiologic Studies Depression Scale
MET: metabolic equivalent of task
PA: physical activity
PCS and MCS: Physical and Mental Health Composite Scores
PSS: Perceived Stress Scale
SF-12: short-form survey
SFA: saturated fatty acids
