# Supplementary material for: A Virtual World Versus Face-to-Face Intervention Format to Promote Diabetes Self-Management Among African American Women: A Pilot Randomized Clinical Trial
Source: JMIR Res Protoc. 2014 Oct 24;3(4):e54. doi: 10.2196/resprot.3412 (PMC4259910; doi:10.2196/resprot.3412)
Supplement: Supplementary file 1 [file resprot_v3i4e54_app1.pdf]

Multimedia Appendix 1

Figure 2 contains pictures of VW intervention sessions and edited clips of the sessions which can be accessed at http://www.youtube.com/watch?v=ThoLohgfGVM.
